# The assessment of walking skills: Italian version

**DOI:** 10.3389/fneur.2025.1579638

**Published:** 2025-05-20

**Authors:** Maria Daniela Cortese, Gianmarco Cernuzio, Paolo Tonin, Konstantinos Priftis, Francesco Piccione

**Affiliations:** ^1^S. Anna Institute, Research in Advanced Neurorehabilitation, Crotone, Italy; ^2^European University of Rome, Rome, Italy; ^3^Department of General Psychology, University of Padua, Padua, Italy; ^4^Unit of Neurorehabilitation, Section of Brain Injury Rehabilitation, Hospital-University of Padua, Padua, Italy

**Keywords:** gait apraxia, assessment of walking skills, apraxia of postural transitions, neuropsychological disorder, diagnostic tools

## Abstract

Apraxia is a neuropsychological disorder that impairs voluntary, purposeful movements, with “Gait apraxia” specifically affecting walking. Because of the lack of standardized diagnostic tools, in Italian, we translated and adapted the “Assessment of Walking Skills” (AWS) scale, originally developed for identifying gait apraxia in Alzheimer’s patients. The AWS includes 42 items that evaluate trunk and leg movements, useful for comparing the performance between patients and healthy controls. This translation and adaptation represent a critical step toward standardizing the AWS scale for the Italian population.

## Introduction

Apraxia encompasses neuropsychological disorders impairing voluntary, purposeful movements, often resulting from left hemisphere lesions. It is not explained by basic sensory or motor deficits like hemiparesis or ataxia (1). The most common form, limb apraxia, includes ideomotor, ideational, and limb-kinetic subtypes, each linked to damage in specific areas of a network involving the frontal and parietal cortex, the basal nuclei, and the white matter fasciculi (2, 3). These subtypes are essential for understanding the diverse impacts of cerebral lesions.

Gait apraxia (4), also known as “apraxia of postural transitions” or “postural transition apraxia” (5, 6), is a less explored apraxia subtype that impairs trunk and limb movements that are necessary for walking. This neurological condition hinders smooth and coordinated movements during the patient’s shifts between body positions. These shifts are critical for actions like standing from a chair or moving from sitting to standing (6–9). Affected patients struggle to initiate and execute these transitions, often displaying hesitant, jerky movements and difficulty maintaining balance (5, 6).

Gait apraxia typically arises from damage to specific brain regions responsible for planning, executing, and coordinating motor movements. Gait apraxia is frequently caused by neurological diseases such as stroke, traumatic brain injury, Parkinson’s disease, or certain types of dementia; it often co-occurs with other types of apraxia. The complexity of gait apraxia requires precise diagnostic tools and an in-depth understanding of the disorder to effectively treat the affected patients (6, 10, 11).

The diagnosis and management of gait apraxia usually involve a comprehensive neurological evaluation, which should include clinical assessments, physical examinations, and possibly neuroimaging (11, 12). To date, however, there are no standardized tools, available in Italy, specifically designed to assess gait apraxia. The lack of such instruments may contribute to the limited diagnosis and reporting of this disorder.

Della Sala et al. (4) conducted a study on patients with mild to moderate Alzheimer’s disease through the “Assessment of Walking Skills” (AWS) scale for evaluating walking and related movements. Della Sala et al. (4) found that 40% of the Alzheimer’s group performed below the cut-off score on this scale. Those results underscored the importance of standardized scales, like the AWS, in detecting gait apraxia and distinguishing it from other causes of walking deficits.

The structure of the AWS scale can be highly useful for evaluating gait apraxia because the AWS provides specific information that is suitable for an accurate differential diagnosis. The objective of the present study was the translation and adaptation of the AWS scale from English to Italian. We aimed to make the AWS scale accessible to Italian-speaking patients, researchers, and healthcare professionals, thereby contributing to a broader understanding and assessment of gait apraxia within the Italian-speaking community.

## Materials and methods

### The AWS scale

The AWS comprises 42 items (plus four items used as introductory examples) designed to evaluate trunk and walking movements. The items included in the final version of the scale were grouped into two components: assessment of trunk movements (22 items) and assessment of walking (20 items), with a scoring range from zero to 42 (4).

Practice items were provided for each of the two components of the scale (trunk vs. walking movements). In selecting the items of the AWS scale (4), the features characterizing gait apraxia were categorized into four groups: inappropriate righting and supporting reactions during the transition from lying and sitting to the upright position; disequilibrium while walking; unnecessary (literally “parasitic”) or dyskinetic movements; locomotor disorders, which include gait initiation failure, irregularities in rhythm, size, speed, and direction of steps, uncoordinated movements of the trunk, lower limbs, and upper arm swinging, as well as an inability to negotiate turns (13).

In translating the AWS to Italian, special emphasis was placed on maintaining linguistic equivalence while adapting the conceptual meaning of the scale (14). With this aim, for our translation we adhered to the WHO protocol for translations (14, 15) to ensure conceptual, semantic, and cultural equivalence between the original and the translated version. To achieve this goal, the following steps were undertaken: (i) two bilingual translators-one an expert in the field and the other a layperson- translated the scale, along with the administration and scoring instructions; (ii) three experts in the field of rehabilitation reviewed the translated scale to ensure conceptual equivalence and terminological coherence with the original version; (iii) the most appropriate version was then back-translated into English, by two bilingual experts in clinical rehabilitation; (iv) finally, the translated items were presented to 10 physiotherapists to assess clarity and practical understanding, requiring them to perform the tasks after hearing the instructions (14).

This rigorous translation process ensured that the Italian version of the AWS faithfully mirrored the content and structure of the original scale, thereby allowing for accurate evaluation of gait apraxia in the Italian context.

### Statistical analysis

Descriptive statistics were used to assess the concordance between the two bilingual translators for the English-to-Italian translations of the AWS scale. The percentage of concordance in the item translations was calculated for all the items of the scale and the two subscales: trunk and walking movements. Additionally, physiotherapists’ understanding and any difficulties interpreting the items were evaluated to ensure clarity in the translated scale.

## Results

The overall concordance for the translated AWS items was 93% (39/42), with discrepancies in three items: two from the walking movements subscale (10%) and one from the trunk movements subscale (4%). These discrepancies derived from linguistic interpretation challenges and from translating nuanced clinical descriptions into Italian. To resolve these issues, a consensus approach involving bilingual experts, including clinicians and translators, was employed to ensure that the translation was clinically accurate and linguistically appropriate. The final item selection prioritized clinical relevance and comprehensibility, while maintaining the scale’s integrity and applicability (Appendix). Given the high concordance and positive feedback from physiotherapists, no further statistical analysis was necessary for the back translation.

## Discussion

We addressed the critical need for standardized tools to assess gait apraxia by translating and adapting the AWS scale into Italian. The translation process achieved a remarkable 93% concordance across items, thereby showing a successful adaptation that respects the original scale, while making it accessible to Italian-speaking patients, clinicians, and researchers.

The availability of the AWS in Italian has profound implications for clinical practice. It enhances diagnostic precision for gait apraxia and enables tailored intervention strategies, thereby improving patient outcomes. The comprehensive evaluation of walking and trunk movements, by using the AWS scale, offers to clinicians a nuanced tool for assessing movement disorders, a crucial step toward personalized rehabilitation paths. Integrating the AWS scale with existing diagnostic frameworks and instrumental assessments could provide a fuller understanding of gait apraxia. Indeed, this integration can unveil the intricate relations between neural damage and motor function, thereby improving therapeutic strategies.

Nevertheless, our study has some limitations. The translation process, although rigorous, highlighted the challenges of achieving conceptual equivalence between the English and the Italian version of the AWS, thereby underscoring the need for further validation studies. Future research should focus on validating the Italian version of the AWS against clinical outcomes and exploring its applicability across diverse neurological conditions. Indeed, the standardization of the Italian version of the AWS, through a well-stratified and adequately large control group of healthy participants, will be the next and final step for rendering the scale fruitful to Italian patients, clinicians, and researchers. Nonetheless, the Italian version of the AWS could be already used to compare the performance of small groups of patients with that of controls, or even to compare the performance of a single patient with that of a small control group.

Reflecting on the cultural and linguistic sensitivities inherent in the translation process has reinforced the importance of adapting clinical tools to meet the needs of various populations of patients. By doing so, we ensure that the AWS is linguistically accurate and culturally resonant with Italian-speaking patients.

## Conclusion

The Italian translation of the AWS represents a significant contribution to neurology and rehabilitation. It offers, indeed, a standardized, culturally-adapted tool for assessing gait apraxia, thereby improving patient care.

## Data Availability

The raw data supporting the conclusions of this article will be made available by the authors, without undue reservation.
