# Supplementary material for: The assessment of walking skills: Italian version
Source: Front Neurol. 2025 May 20;16:1579638. doi: 10.3389/fneur.2025.1579638 (PMC12129749; doi:10.3389/fneur.2025.1579638)
Supplement: Supplementary file 1 [file Supplementary_file_1.docx]

**ASSESSMENT OF WALKING SKILLS (AWS)**

| Cognome e Nome: | Data di nascita: Età: | Data di esecuzione: |
| --- | --- | --- |

| **Numero** | **DEAMBULAZIONE** | **Fallito** | **Eseguito** |
| --- | --- | --- | --- |
| **Esempio** | Camminare con la mano destra sull’addome e la mano sinistra sulla schiena |  |  |
| **Esempio** | Camminare con la mano sinistra sull’addome e la mano destra sulla schiena |  |  |
| 1 | Camminare sul posto |  |  |
| 2 | Camminare in avanti |  |  |
| 3 | Camminare su base allargata |  |  |
| 4 | Effettuare passi laterali verso destra guardando in avanti |  |  |
| 5 | Camminare in linea retta mettendo un piede davanti l’altro |  |  |
| 6 | Camminare all'indietro |  |  |
| 7 | Camminare con la punta dei piedi verso l’esterno |  |  |
| 8 | Effettuare passi laterali verso sinistra guardando in avanti |  |  |
| 9 | Camminare sui talloni |  |  |
| 10 | Camminare con la punta dei piedi verso l’interno |  |  |
| 11 | Camminare con il tronco inclinato a sinistra |  |  |
| 12 | Camminare con l’arto superiore sinistro esteso verso l’alto e l’arto destro penzolante |  |  |
| 13 | Camminare con le braccia conserte |  |  |
| 14 | Camminare con il tronco inclinato in avanti |  |  |
| 15 | Camminare con l’arto superiore di destra esteso verso l’alto e il braccio sinistro penzolante |  |  |
| 16 | Camminare con il tronco inclinato a destra |  |  |
| 17 | Camminare con le mani dietro la nuca |  |  |
| 18 | Camminare con gli arti fermi (senza oscillare) lungo il tronco |  |  |
| 19 | Camminare con il tronco inclinato all'indietro |  |  |
| 20 | Camminare superando un ostacolo immaginario (sul pavimento) |  |  |
|  |  |  |  |
|  | **CONTROLLO DEL TRONCO E PASSAGGI POSTURALI** |  |  |
| **Esempio** | Sedersi (a bordo destro del letto) |  |  |
| **Esempio** | Sedersi a bordo del letto |  |  |
| 21 | Flettere in avanti il capo |  |  |
| 22 | Ruotare la testa verso sinistra |  |  |
| 23 | Flettere lateralmente a destra il capo |  |  |
| 24 | Estendere indietro il capo |  |  |
| 25 | Ruotare il capo a destra |  |  |
| 26 | Flettere lateralmente il capo a sinistra |  |  |
| 27 | Flettere il tronco in avanti |  |  |
| 28 | Sollevare le spalle |  |  |
| 29 | Tirare l’addome in dentro |  |  |
| 30 | Inclinare lateralmente il tronco a sinistra |  |  |
| 31 | Inclinare il tronco indietro |  |  |
| 32 | Inclinare anteriormente, a destra (o sinistra), il tronco |  |  |
| 33 | Spingere l’addome in fuori |  |  |
| 34 | Sedersi (dalla posizione in piedi) |  |  |
| 35 | Sdraiarsi supino (partendo dal lato destro del letto) |  |  |
| 36 | Sdraiarsi prono (partendo dal lato destro del letto) |  |  |
| 37 | Girarsi da prono a supino |  |  |
| 38 | Girarsi da supino a prono |  |  |
| 39 | Da prono, alzarsi |  |  |
| 40 | Sdraiarsi supino (partendo dal lato sinistro del letto) |  |  |
| 41 | Mentre si è supini, flettere e abbracciare entrambe le ginocchia |  |  |
| 42 | Sdraiarsi prono (partendo dal lato sinistro del letto) |  |  |
| **Punteggio totale**: | | | |

ISTRUZIONI PER LA SOMMINISTRAZIONE

Sono presenti due valutatori (uno mostra i movimenti al paziente l’altro interagisce osserva, e registra la prestazione).

Dopo aver spiegato il compito assicurarsi che il paziente abbia compreso e appreso la consegna per l’esecuzione

“*Adesso le mostrerò alcuni movimenti che lei dovrà ripetere così come li ho eseguiti io*”

Il punteggio da assegnare è **0** = *esecuzione fallita*, **1** = *esecuzione corretta*

*Esecuzione fallita*:
